# „*Ich stehe jeden Dienst mit mir selbst im Konflikt*“–„Moral distress“ bei Altenpflegenden während der COVID-19-Pandemie

**DOI:** 10.1007/s16024-022-00366-2

**Published:** 2022-01-31

**Authors:** A. Begerow, U. Gaidys

**Affiliations:** Department Pflege und Management, Hochschule für Angewandte Wissenschaften Hamburg, Alexanderstr. 1, 20099 Hamburg, Deutschland

**Keywords:** Moralische Dilemma, Pflegeheime, Pandemie, Qualitativer Survey, Moral dilemma, Nursing homes, Pandemic, Qualitative survey

## Abstract

**Hintergrund:**

Aufgrund der COVID-Pandemie-bedingten Veränderungen sind Pflegende im Setting der stationären Altenpflege besonderen Belastungen ausgesetzt und können in Bezug auf ihre moralische Verantwortung Dilemmata erleben.

**Ziel:**

Ziel dieser Untersuchung ist es, die Auswirkungen und Wahrnehmungen hinsichtlich der Entstehung von Moral distress von Pflegenden im Setting der Altenpflege während der SARS-CoV-2-Pandemie in Deutschland zu explizieren und daraus Schlussfolgerungen für die Gestaltung von Pflege in stationären Pflegeeinrichtungen zu ziehen.

**Methode:**

Auf der Basis einer Subgruppenanalyse (*n* = 510) des qualitativen Surveys der COVID-19-Pflegestudie werden Wahrnehmungen von Altenpflegenden während der Pandemie in Deutschland dargestellt und hinsichtlich der Entwicklung von Moral distress ausgewertet.

**Ergebnisse:**

Durch die Analyse können 5 Kategorien („*Dass wir keine Zeit haben, um ordentlich pflegen zu können*“, „*Vereinsamung*“, „*Konflikte mit Angehörigen und Bewohnern*“, *„Ständige Angst um Patientensicherheit, aber auch um die eigene Sicherheit“* und „*Trauer, Stress und Wut*“) identifiziert werden, die differenzierte Kriterien zum Entstehen von Moral distress bei den Altenpflegenden sowie deren Auswirkungen darlegen.

**Schlussfolgerungen:**

Aus den Erkenntnissen ist zu schlussfolgern, dass strukturelle und fachliche Lösungen entwickelt werden müssen, die es Pflegenden ermöglichen, ihr eigenes Arbeitsumfeld zu gestalten und fachliche Versorgungsentscheidungen selbstständig zu übernehmen.

## Abstract (en)

**Background:**

Due to the COVID-19 pandemic-related changes and guidelines, nurses in the setting of inpatient geriatric care are exposed to specific burdens and may experience dilemmas with respect to their moral responsibility.

**Aim:**

The aim of this study was to explicate the effects and perceptions regarding the development of moral distress of nurses in the setting of geriatric care during the SARS-CoV‑2 pandemic in Germany and to draw conclusions for the design of care in residential care settings.

**Methods:**

Based on a subgroup analysis (*n* = 510) of the qualitative survey of the COVID-19 Nursing Study, perceptions of nurses working in residential care settings during the pandemic are presented and evaluated with respect to the development of moral distress.

**Results:**

The results show five categories *(“That we have no time to care properly”, “Loneliness”, “Conflicts with relatives and residents”, “Constant fear for patient safety but also for one’s own safety”* and “*Grief, stress and anger”*), which represent differentiated criteria for the development of moral distress among nurses as well as its effects.

**Conclusion:**

It can be concluded that structural and professional solutions must be developed that enable nurses to shape their own working environment and to undertake professional nursing decision-making competence.

## Hintergrund

Aufgrund der COVID-19-Pandemie wurden zum Schutz vulnerabler Personengruppen, die in Pflegeeinrichtungen leben, besondere Hygiene- und Isolationsmaßnahmen erlassen. Beispielsweise wurde ein bundesweites Besuchsverbot veranlasst (BIVA 2020), das einerseits Fürsorge und Schutz ermöglichen soll, andererseits eine Isolation der Pflegebedürftigen darstellt, die mit negativen gesundheitlichen Folgen einhergehen kann (Fischer et al. 2020).

Vor dem Hintergrund solcher pandemiebedingten Richtlinien können Pflegende in Pflegeeinrichtungen Konflikte in Bezug auf ihre moralische Verantwortung erleben. Pflegende haben die professionelle Verpflichtung, ihr Handeln nach den Prinzipien der Selbstbestimmung, dem Wohltun, der Schadensvermeidung und der Gerechtigkeit für die Pflegebedürftigen auszurichten (Beauchamp und Childress 2019). Diese Prinzipien können allerdings im Widerspruch zu den pandemiespezifischen Schutz- und Isolationsstrategien für ältere Menschen in Pflegeeinrichtungen stehen. Wenn Pflegende wissen, welche Maßnahmen aus berufsethischer und professionell-fachlicher Perspektive richtig sind, sich aber aus verschiedenen Gründen nicht in der Lage fühlen, diesen Prinzipien entsprechend, eine angemessene Vorgehensweise zu ergreifen oder Schaden abzuwenden, erleben sie moralischen Stress – auch „moral distress“ genannt (Epstein und Hamric 2009; Kleinknecht-Dolf et al. 2015; Spirig et al. 2014; Hamric und Epstein 2017).

Zusätzlich zu pandemiebedingten externen Richtlinien und gesetzlichen Vorgaben gelten negative institutionelle Faktoren, etwa eine qualitativ und quantitativ schlechte Personalbesetzung, die angemessene Pflege vermindert, als begünstigend für Moral distress (Austin et al. 2017; Wolf et al. 2016; Barth et al. 2019; Lazzari et al. 2020). Zudem beeinflussen individuelle intrinsische Faktoren Moral distress (Cacchione 2020). So können subjektive Wahrnehmungen hinsichtlich der eigenen Rolle während der pandemischen „Extremsituation“ oder auch die persönlichen Bewältigungsfähigkeiten das Erleben von Moral distress beeinflussen (Cacchione 2020).

Ungelöster Moral distress wird bei Pflegenden mit Gefühlen wie Wut, Frustration, mit Schuldempfinden, einem Verlust des Selbstwertgefühls, mit Trauer, Angst und Albträumen, Motivationslosigkeit, emotionaler Erschöpfung, aber auch mit Depressionen und Burn-out sowie verminderter moralischer Sensibilität oder Depersonalisation in Verbindung gebracht (Hanna 2004; Wiegand und Funk 2012; Rushton 2017; Nasrabadi et al. 2018). Überdies gilt Moral distress manchen Pflegefachpersonen als Grund dafür, ihren Arbeitsplatz oder sogar den Pflegeberuf zu verlassen (Rushton 2006; Corley et al. 2005; Rittenmeyer und Huffman 2009; Laurs et al. 2020; Lazzari et al. 2020).

Trotz der bekannten pandemiebedingten massiven Belastungssituation im Bereich der stationären Altenpflege und der gravierenden Auswirkungen von Moral distress scheinen die Altenpflegenden bisher keine besondere Berücksichtigung im pflegewissenschaftlichen Diskurs zu finden. Dies ist erstaunlich, da settingabhängige Merkmale der stationären Altenpflege, wie die fehlende dauerhafte ärztliche Anwesenheit oder die engen Beziehungen zu den Pflegebedürftigen und die häufige Konfrontation mit dem Sterben dieser, als bedeutsam für die Entwicklung von Moral distress gelten (Kada und Lesnik 2019). Demzufolge müssen sowohl kurz- als auch langfristige Unterstützungsangebote zur Bewältigung von Moral distress während der Pandemie für Pflegende in der stationären Altenpflege diskutiert und entwickelt werden. Damit sich solche Angebote an den Bedürfnissen Altenpflegender orientieren, werden in diesem Beitrag folgende Fragestellungen bearbeitet:

- Welche situativen Pandemiefaktoren lösen Moral distress bei Altenpflegenden während der Pandemie aus und verstärken diesen?
- Welche Auswirkungen hat dies auf die Situation der Altenpflege?

## Ziel

Ziel dieser Untersuchung ist es, die Auswirkungen und Wahrnehmungen hinsichtlich der Entstehung von Moral distress von Pflegenden im Setting der Altenpflege während der SARS-CoV-2-Pandemie in Deutschland zu explizieren. Aus diesen Ergebnissen sollen Schlussfolgerungen für Entwicklungen von fachlichen, strukturellen und institutionellen Rahmenbedingungen gezogen werden, die moralisches Handeln in der Versorgung von älteren Menschen ermöglichen.

## Methode

Zur Abbildung der subjektiven Wahrnehmungen von Altenpflegenden während der Pandemie wurde ein qualitatives Forschungsvorgehen angewendet (Lamnek 2010). Die Datenerhebung erfolgte mittels webbasiertem qualitativem Survey nach Jansen (2010), indem durch offen formulierte Leitfragen alle Studienteilnehmenden angeregt wurden, ihre Wahrnehmungen und Erfahrungen während der Pandemie als Narrative zu verschriftlichen. Die Leifragen umfassten die Themen der Veränderung in der Bewohner*innenversorgung, der Wahrnehmung von Versorgungsqualität während der Pandemiesituation, der Auswirkungen der Pandemie im Arbeitsalltag und der Belastungserfahrungen in der Pandemie. Zusätzlich wurden das Arbeitssetting und die Jahre im Beruf erfragt. Vorab erfolgte ein ethisches Clearing durch die zuständige Ethikkommission. Die hier berichteten Ergebnisse beruhen auf einer Subgruppenanalyse der COVID-19-Pflegestudie zum Erleben von Pflegenden während der SARS-CoV-2-Pandemie (Begerow und Gaidys 2020a). In der Studie wurden im Zeitraum von April 2020 bis Februar 2021 ausschließlich Pflegende (*n* *=* 3424) mittels eines webbasierten qualitativen Surveys zeitlich kongruent mit den ersten 2 COVID-19-Wellen befragt. Der Link zur Studie wurde deutschlandweit über soziale Medien, Berufsorganisationen und über Hochschulverteiler veröffentlicht. Damit wurde eine Gelegenheitsstichprobe realisiert. Auf dieser Datengrundlage erfolgte eine Subgruppenanalyse, die Pflegende (*n* = 510), die angegeben haben, im Setting der Altenpflege tätig zu sein, einschloss. Die zusammenfassende Inhaltsanalyse erfolgte nach Mayring (2015) unter Verwendung der Software MAXQDA (ein Produkt der VERBI GmbH, Berlin, Deutschland) 2018 in 7 Schritten (Bestimmung der Analyseeinheiten, Paraphrasierung, Generalisierung der Paraphrasen, Reduktion durch Selektion, Reduktion durch Bündelung, Zusammenstellung der neuen Aussagen als Kategoriensystem, Rücküberprüfung am Ausgangsmaterial). Diese Analyse ist für die Verarbeitung von großen Datenmengen, wie sie durch die Narrative generiert wurden, besonders geeignet. Die Datenmenge wurde auf ein überschaubares Maß reduziert, wobei die prägnanten Aussagen und Inhalte des Ausgangsmaterials erhalten blieben (Mayring 2015). Die Kategorienbildung erfolgte induktiv, die Aussagen der Teilnehmenden wurden als Validierung der Kategorien verwendet. Für die Studie sind die qualitativen Gütekriterien Glaubwürdigkeit, Übertragbarkeit und Verlässlichkeit nach Lincoln und Guba (1994) leitend. Die Glaubwürdigkeit der Ergebnisse ist durch die Darstellung der wörtlich zitierten schriftlichen Aussagen der Pflegenden gegeben und die Übertragbarkeit der Erkenntnisse durch die Übereinstimmungen, die die Narrative der Pflegenden über alle Settings hinweg aufweisen (Begerow et al. 2020; Begerow und Gaidys 2020b). Die Ergebnisse scheinen verlässlich zu sein, da die Verschriftlichungen der Pflegenden eine hohe Kohärenz über den beobachteten Pandemieverlauf aufweisen.

## Ergebnisse

Hier werden die Ergebnisse der Subgruppenanalyse von Pflegenden im Setting der stationären Altenpflege (*n* = 510) dargestellt, die während der zweiten Welle im Zeitraum vom 31. Oktober 2020 bis 25. Februar 2020 an der COVID-19-Pflegestudie teilgenommen haben. Es werden 5 Kategorien identifiziert, die thematisch eng miteinander verbunden sind. Das Thema des Personal- und damit Zeitmangels („*Dass wir keine Zeit haben, um ordentlich pflegen zu können*“) ist dabei für die Entstehung von Moral distress von überragender Bedeutung und wird auch in den weiteren Kategorien* (*„*Vereinsamung*“ – „*das Personal leidet, die Bewohner so zu sehen*“, „*Konflikte mit Angehörigen und Bewohner *[…] *Andere Berufsgruppen kollidieren mit uns*“, *„Ständige Angst um Patientensicherheit, aber auch um die eigene Sicherheit“ *und „*Trauer, Stress und Wut*“) implizit und explizit benannt*. *Nachfolgend werden die Ergebnisse mit wörtlichen Zitaten aus den schriftlichen Narrativen dargestellt, damit die Altenpflegenden selbst zu Wort kommen können.

### *„Dass wir keine Zeit haben, um ordentlich pflegen zu können“*

Pflegende, die in der Altenpflege tätig sind, beschreiben durchgehend in den Narrativen, dass es während der Pandemiesituation zu wenig Pflegende für die zu versorgenden alten Menschen gibt. „*Weniger Personal, mehr Arbeitsaufwand unter harten Bedingungen*“ (MC25). Es werden enorme pandemiebedingte Arbeitsveränderungen, -verdichtungen und die Übernahme pflegeferner Aufgaben beschrieben, wie „*Besuche müssen telefonisch vereinbart und terminiert werden. Mitarbeiter muss für den Eingang abgezogen werden. Kontrolle Platzzuweisungen. Visiten finden teils nicht mehr statt. Quarantäne und Isolierungsregelungen. Zimmerservice, da sich Abstandsregelungen in den Gemeinschaftsräumen nicht umsetzen lassen“*; dies führt dazu, dass „*man keine Zeit mehr hat, sich um die Bewohner zu kümmern und ständig die Station verlassen muss, um Besucher zu testen, und anschließend immer das Zimmer desinfizierend reinigen müssen. Wir sind nachmittags im Spätdienst nur zu zweit und sollen das alles nebenbei managen*“ (GC28). Das Problem des Zeit- und Personalmangels wird durch pandemiebedingte Personalausfälle verstärkt: „*Wir hatten kaum noch Kollegen, da 75* *% des gesamten Personals in Quarantäne waren*“ (MC30). Als Folge arbeiten Altenpflegende beispielsweise „*derzeit mit einer Fachkraft und 2 Pflegehelfern auf 2 Etagen mit je 25 Patienten. Das hat für mich nichts mehr mit Pflege zu tun, das nenne ich nur noch Massenabfertigung!!*“ (DC5). Es wird deutlich, dass infolge des in der Pandemie gestiegenen Personalmangels keine adäquate Pflege zu leisten ist – kurz: „*Personalmangel* *=* *Mangel an Pflegequalität“* (DC5). Die unzureichende Personalbesetzung geht einher mit massiven Einschränkungen, wie „*Keine Zeit für mehr die wirklich wichtigeren Dinge zu haben, wie die Fürsorge, durch auch hohen zeitlichen Druck *[…] *Körperpflege bzw. deren Nachsorgen, oft können kleinere Dinge nicht durchgeführt werden*“ (MC31). Zu „*wenig Zeit für Bewohner*“ (DD13) zu haben, führt zur Klient*innengefährdung mit schwerwiegenden Folgen, wie beispielsweise dem Ausfall von „*Toilettengängen und daraus resultierenden Wunden*“ (GB2).

Durch die unzureichende pflegerische Versorgung der vulnerablen Klient*innengruppe erkennen die Pflegenden ein ethisches Problem, können allerdings infolge des Personal- und Zeitmangels nicht angemessen reagieren, was Moral distress auslöst: „[besonders belastet mich] *dass wir keine Zeit haben, um ordentlich pflegen zu können*“ (JC132) oder auch „[besonders belastet mich] *dass man keine professionelle Pflege durchführen kann. Man mit den Kräften am Limit geht. Keine Zeit für Pat. hat*“ (JC142).

Altenpflegende müssen sich den Rahmenbedingungen während der Pandemie ergeben und können bei der Versorgung der Pflegebedürftigen ihren moralischen und ethischen Werten nicht folgen. Demzufolge erleben die Altenpflegenden eine drastische und belastende Beeinträchtigung der eigenen Handlungsmöglichkeiten, denn „*die Situation in der Pflege ist grauenhaft und wird durch Corona nur noch verschlimmert. Der Personalmangel wird immer deutlicher, und wir haben immer weniger Zeit für unsere Bewohner und das, obwohl sie uns jetzt gerade so sehr brauchen, da sie kaum Besuch bekommen dürfen. Wir sind die Einzigen, die sie mal in den Arm nehmen können, die Einzigen, die ihnen die Hand halten dürfen, wenn sie einschlafen, wir sind die Letzten, die sie sehen, und dennoch haben wir für all das Zwischenmenschliche keine Zeit mehr. Das ist das, was mich persönlich extrem belastet und wodurch meine Motivation einerseits steigt, da ich sie nicht alleine lassen kann, andererseits aber auch sinkt, da ich diese Zeit nicht mehr habe und es einfach kein Ende bzw. eine Besserung in Sicht ist*“ (HC7).

Die unzureichende pflegerische Versorgung führt dazu, dass die Pflegenden keine Möglichkeit haben, nach beruflichen Werten und Standards zu handeln, wie *„Aktivierende Pflege durchzuführen, dazu ist an vielen Tagen keine Zeit mehr! Wenn wir Patienten verlegen müssen, wenn sie COVID-positiv sind, dann bleiben alle anderen auf der Strecke. Es frustriert mich massiv, meine Patienten nicht mehr so zu versorgen zu können, wie vor Corona“ *(JC57). Es kommt zum Verlust ihrer Authentizität sowie zur Deprofessionalisierung: „*Es geschieht ein Rückschritt zur Funktionspflege, spezielle Konzepte gerade zur Betreuung von alten Menschen mit gerontopsychiatrischen Veränderungen finden keine Anwendungen mehr*“ (KE14)*. *Die pflegerische Versorgung der betagten Menschen wird als „*miserabel, warm, satt, sauber, nicht mehr*“ (QD5) beschrieben.

Die additiven Aspekte sowohl der Rahmenbedingungen als auch der wahrgenommenen verminderten Versorgungsqualität führen in der Konsequenz zu moralischem Stress. Die Pflegenden befinden sich in einem Dilemma, denn sie müssen entscheiden, wen, wie und in welchem Umfang sie sich um einzelne Pflegebedürftige kümmern. „*Es ist anstrengend. Ein Zwiespalt, den Bewohnern ihren Alltag so zu gestalten, dass er schön ist, aber trotzdem 20 Bewohner am Tag würdevoll pflegen. Man ist am Limit*“ (BD66). Im Zusammenhang mit Zeit- und Personalmangel kommt es nicht allein durch die Notwendigkeit von Priorisierungen, sondern auch durch einen erlebten Rationierungszwang zu moralischen Problemen: „*Wir müssen immer mehr abknapsen. Einiges an Pflege findet gar nicht mehr statt*“ (JC155).

Es werden moralisch schwierige Situationen beschrieben, da Altenpflegende sich pandemiebedingt gezwungen sehen, gegen ihre moralischen Werte zu handeln. „*Ich fühle mich seit Corona nicht mehr wohl in dem Beruf *[…] *ich habe den Beruf geliebt, nur jetzt durch Corona wird er immer unattraktiver, ich liebe es, an Menschen und mit Menschen zu arbeiten, ihnen zu helfen, nun ist man in dem Job nur noch dafür da, um kein Corona ins Haus zu lassen, alle zu testen und keine Zeit mehr zu haben, die Bewohner gehen ein, Betreuungskräfte machen weniger wie vorher, was für die Bewohner kaum noch Beschäftigung haben *[bedeutet]*, die meiste Zeit nur die Besuchstermine im Blick, die Menschen gehen ein, ob Bewohner oder die Mitarbeiter, es ist für beide Parteien keine schöne und angenehme Situation*“ (OD15).

Die strukturelle Überlastung der Pflegenden durch zu wenige Kolleg*innen, pandemiebedingte Personalausfälle und die Übernahme von pflegefremden Tätigkeiten, führt zu moralischen Belastungssituationen, da die Pflegenden schlicht keine Zeitressourcen haben, um professionelle Pflege durchzuführen. Sie sind entgegen ihrem Professionsverständnis gezwungen, originäre Pflegetätigkeiten, wie Körperpflege, Toilettengänge, Kommunikation zu rationieren und sie sind direkt mit den Konsequenzen dieser Rationierungen, einer für sie unakzeptablen Pflegequalität, konfrontiert.

### *„Vereinsamung*“ – „*Das Personal leidet, die Bewohner so zu sehen*“

Durch die Notwendigkeit zur Reduzierung des direkten Kontakts zu Pflegebedürftigen und dem angewiesenen Besuchsverbot zum Schutz der vulnerablen Personengruppe sind „*Unsere Bewohner *[…] *meistens traurig, ohne Familie. Wir dürfen nicht, wie damals, zusammen Kaffee trinken, uns umarmen und und …*“ (GB20). Pflegende können den pflegebedürftigen alten Menschen keine soziale Teilhabe ermöglichen, und Pflege findet auf Distanz statt: „*die Nähe zu meinen Bewohnern ist ganz wichtig, jetzt muss ich dies bis auf wenige Ausnahmen sein lassen. Umarmungen wie auch an die Hand nehmen etc.*“ (JC12). Ferner beeinträchtigen Schutzausrüstung sowie pandemiebedingte Auflagen die gewohnten Pflegeabläufe und die Wirksamkeit der Pflege. „*Die behinderte Kommunikationsfähigkeit, insbesondere in der Arbeit mit demenziell erkrankten Personen*“ (JD2)*. *Aufgrund der Pandemieauflagen sind Pflegende gezwungen, entgegen der beruflichen Integrität zu handeln: „[besonders belastet mich] *die traurigen Bewohner, für die man sich nicht umfassend sorgen kann, weil man nicht die Zeit hat, um sich mal 20* *min mit jedem Bewohner hinzusetzen, um sie wieder bei Laune zu halten*“ (IB1).

Infolgedessen führt das pandemiebedingte Besuchsverbot die Pflegenden in moralisch schwierige Situationen, denn* „*[besonders belastet mich,] *dass die Bewohner unter dem Nicht-Sehen der Angehörigen leiden. Die Zeit und die Pflegekräfte fehlen, dieses ausgleichen zu können und für die Bewohner da zu sein“ *(JC154). Diese Situation ist belastend und erscheint unlösbar: „*die Bewohner leiden, das Personal leidet, die Bewohner so zu sehen und nichts ändern zu können*“ (JC77). Pflegende erleben eine Hilflosigkeit und eine „*ständige Angst um Bewohner, kaum Möglichkeit der Beschäftigung, keine Besuche erlaubt, hohe Einsamkeit*“ (PB6). Es wird deutlich, dass der moralische Stress der Pflegenden mit dem Leiden der Pflegebedürftigen verbunden ist: „*Für die Bewohner finde ich es einfach belastend, keine Angehörigen mehr empfangen zu können, immer wiederkehrende Quarantäneregelungen. Essen alleine, den ganzen Tag alleine auf dem Zimmer sein, besonders für demente Personen. Weniger Mobilisation, weniger Ansprache. Eine Bewohnerin meinte einmal, wenn mich nicht das Virus erwischt, dann bringt mich die Einsamkeit um*“ (CD50). Das Besuchsverbot wird von den Pflegenden wahrgenommen als „*Vereinsamung, die Leute haben Angst und sind unsicher*“ (MD8). Altenpflegende wissen um eine ethisch angemessene pflegerische Handlung in dieser Situation, sind aber nicht in der Lage, entsprechend zu handeln, denn „*Das Pflegepersonal kann die psychischen Belastungen nicht auffangen*“ (JC15). Es gibt keine adäquaten Lösungen, dem Besuchsverbot und der damit verbundenen Isolation der Pflegebedürftigen entgegenzuwirken, was wiederum eine Quelle moralischer Bedrängnis für die Pflegenden darstellt. „*Dass kein Besuch kommt, ist für uns sehr schwierig, ich sage schon nicht mehr, ich gehe heim*“ (GB15). Eine besondere moralische Problematik des Besuchsverbots für Pflegende ist, dass Pflegebedürftige sterben, ohne dass ihre An- und Zugehörigen anwesend sein dürfen. Pflegende beschreiben es als belastend, wenn „*Menschen *[…] *allein sterben, ohne nochmal ihre Angehörigen gesehen zu haben*“ (IB6). Dazu kommt, „*Das Sterben der Menschen, es ist kein ‚normales‘ Sterben, die Menschen leiden, sind wesensverändert, kein Geschmack, kein Geruch und somit keinen Appetit, Angst in den Augen der Patienten/Bewohner. Angehörige zeigen wenig Verständnis, das Virus wird verleugnet*“ (MD25). Das Besuchsverbot birgt ein weiteres Dilemma, wie nachfolgend beschrieben „*Mein größter Konflikt ist das Besuchsverbot, wir haben es gelockert, so dass jeder Patient mind. einmal Besuch bekommen kann, jedoch halten Angehörige sich dann nicht an Absprachen und gefährden ihre eigene Familie und uns als Pflegepersonal*“ (JD130). Die fehlenden Interaktionsmöglichkeiten der Bewohner*innen mit ihren Angehörigen, aber auch mit den Pflegenden, sind für die Studienteilnehmenden eine moralische und ethische Konfliktsituation, da Interaktion und Beziehungsgestaltung wesentliche therapeutische Merkmale pflegerischer Arbeit darstellen.

### *„Konflikte mit Angehörigen und Bewohnern *[…]. *Andere Berufsgruppen kollidieren mit uns“*

Der Einbezug von Zu- und Angehörigen ist ein wichtiger Bestandteil der Pflege, doch durch notwendige Schutz- und Hygieneauflagen wird dieser enorm eingeschränkt. Es kommt zu Dissonanzen und Unstimmigkeiten mit Angehörigen, dabei sind „*Die Angehörigen *[…] *zum Teil vorwurfsvoll und man hat oft das Gefühl, dass wir an der Pandemie schuld seien*“ (MD21). Das Erleben von Schuldzuweisungen und Vorhaltungen durch Angehörige bei gleichzeitiger Sorge um die Pflegebedürftigen ist schwer zu ertragen: „*Es tut mir weh zu sehen, wie wir die Senioren regelrecht isolieren, vor ihren Angehörigen, Bewohnern mit einer demenziellen Erkrankung kann es dadurch auch schneller passieren, dass sie ihre Angehörigen nicht mehr erkennen, und wir als Pflegepersonal müssen dann meist die Schuld dafür tragen, weil Angehörige keinen anderen schuldig sehen als uns*“ (JC25). Unstimmigkeiten und Konflikte mit Angehörigen in Kombination mit Missachtung von Autonomie, Bedürfnissen und Sicherheit der ihnen anvertrauten Menschen führt bei Pflegenden zu Hilflosigkeit und Unverständnis: „*Meine Bewohnerinnen und Bewohner haben Angst vor dem Virus und daran zu erkranken. Ich versuche, so gut ich kann, alle Maßnahmen bei uns in der Einrichtung umzusetzen und viel Aufklärungsarbeit gegenüber Angehörigen zu leisten. Leider gibt es eine Vielzahl an Angehörigen, deren Unmut gegenüber den eingeschränkten Besuchen so hoch ist, dass sie mit Beschwerden gegenüber der Heimaufsicht immer wieder dafür sorgen, dass Besuche stattfinden müssen, wo ich nicht gewährleisten kann, dass ein Besuch hygienisch korrekt abläuft. Diese Hilflosigkeit belastet mich derzeit sehr, da ich nicht verstehen kann, wie Behörden und Institutionen sich derart in solche Angelegenheiten mischen können. Wir sind es, die mit bestem Wissen und Gewissen an der Basis kämpfen, und versuchen, alles dafür zu tun, unter Berücksichtigung aller Hygienemaßnahmen soziale Kontakte aufrechtzuerhalten. Warum können diese Institutionen uns in solch einer Zeit nicht vielmehr den Rücken stärken? Das belastet mich besonders in dieser Zeit sehr*“ (OC12). Deutlich wird zudem die moralische Bedrängnis der Pflegenden aufgrund hierarchischer Kontroversen und institutioneller Strukturen, die ein autonomes und professionelles Handeln nach eigenen moralischen Vorstellungen verhindern.

Überdies werden pandemieassoziierte Konflikte mit den Pflegebedürftigen selbst und mit anderen Professionen beschrieben:* „Konflikte mit Angehörigen und Bewohnern aufgrund des stetigen Wechsels der Auflagen. Andere Berufsgruppen kollidieren mit uns*“ (JC162). In den stationären Pflegeeinrichtungen, so wird es von den Studienteilnehmenden berichtet, kommt es zu eingeschränkter medizinischer Versorgung durch „*Ärzte, die das Heim nicht betreten wollen, weil sie keine Lust haben, sich testen zu lassen*“ (JA39). Pflegende nehmen die hohe Verantwortung für die Pflegebedürftigen bei geringem Handlungsspielraum als sehr herausfordernd wahr: „*erschwerter Handlungsspielraum bei Notfällen *[…] höheres Verantwortungsgefühl, wenn man Patient*innen ins Krankenhaus schicken muss, (stärkeres Abwägen) aus Angst, dass sie sich dort infizieren“ (FC19). Sie fühlen sich mit ethischen Entscheidungen allein gelassen. Gleichzeitig berichten Pflegende von fehlenden Entscheidungsmöglichkeiten und unangemessener Unterstützung, um die Pandemiesituation zu bewältigen. „*Die vielen Sterbefälle ertragen/verarbeiten. Kaum Zeit dazu, da ständig neue Notfälle und Herausforderungen. Krankenhauseinweisung von positiven Bewohnern, wenn diese erst am ‚Ersticken‘ sind. Vorher geschieht keine Einweisung durch Notarzt. Neue medizinische Herausforderungen fürs Personal*“ (BC45). Die interprofessionelle Zusammenarbeit wird v. a. dann als belastend erlebt, wenn die pflegerische Expertise infrage gestellt wird oder sich die Versorgung allein auf COVID-19 fokussiert und damit eine Depriorisierung von Nicht-COVID-19-Infizierten erfolgt, wie im nächsten Beispiel aufgezeigt wird: „*Ich kann nicht mehr … viele Bewohner können nicht mehr …Die ganzen Auflagen und Richtlinien, die sich sehr oft kurzfristig ändern, machen mich fertig. Bei mir ist in der Nachtschicht ein Bewohner gestürzt, er hatte eine Kopfplatzwunde und hat stark geblutet. Ich habe einen Krankenwagen gerufen. Als sie ankamen, war ihr erster Schritt, dem Bewohner die Temperatur zu messen und zu fragen, ob bei ihm bezüglich Corona bekannt wäre oder bei einem andern Bewohner im Haus. Das muss man sich mal vorstellen: Da liegt jemand stark blutend, und man muss erstmal die Temperatur messen …*“ (AB21). Auch durch strikte und immerfort der aktuellen Pandemiesituation anpasste Arbeitsanweisungen können Pflegebedürftige nicht individuell und bedürfnisangepasst versorgt werden, was zu Moral distress bei den Pflegenden führt. Institutionelle Anordnungen, die sich entgegen der eigenen moralischen Auffassungen im Umgang mit den Pflegebedürftigen richten, wie „*Es soll nur noch das Nötigste gemacht werden*“ (JC77) oder „*Es werden schwach positive mit negativen Patienten zusammengelegt, obwohl die Klinik gerade voll mit Corona war. Es wird auf Post-COVID-Mitarbeiter keine Rücksicht genommen. Ängste werden nicht ernst genommen*“ (OD18), fehlende Unterstützung und respektloser Umgang durch Führungskräfte, die zu ethischen Konflikten führen, z. B. „*Wenn ein Kollege positiv ist, wird man abgehalten, Dinge zu erzählen, um nicht selbst in Quarantäne zu kommen*“, kann eine Grundlage für Moral distress darstellen. Zudem werden eine Zunahme an mangelnder Kollegialität „*irgendwie fehlt der Rückhalt durch das Team*“ (JE12) und vermehrt „*Konflikte im Team*“ (EE4) beschrieben, die zusätzlich belastend wirken.

In den Aussagen der Pflegenden zeigt sich eine Veränderung ihrer professionellen Rolle gegenüber Angehörigen. An- und Zugehörige sind ein bedeutsamer Teil einer professionellen Beziehungsgestaltung, zugleich sind diese immer auch Klient*innen der Pflege. In der Pandemiesituation verstärkt sich allerdings die administrativ-amtliche Rolle der Pflegenden gegenüber den Angehörigen. Pflegende sollen offizielle und zudem noch ständig wechselnde Anordnungen gegenüber den An- und Zugehörigen kommunizieren und umsetzen. Ebenfalls ist in den Aussagen ersichtlich, dass Pflegende sich administrativen Entscheidungen ausgeliefert sehen, die ihrer professionell situativen Einschätzung entgegenstehen.

### *„Ständige Angst um Patientensicherheit, aber auch um die eigene Sicherheit“*

Pflegende beschreiben einen „*Konflikt zwischen eigener Gesundheit und deren der Pflegebedürftigen*“ (LC2). Sie erleben ein Dilemma zwischen persönlicher Sicherheit und beruflicher Integrität sowie beruflicher und persönlicher Verantwortung. Dies zeigt sich durch „*Ständige Angst um Patientensicherheit, aber auch um die eigene Sicherheit und die der Familie*“ (JC25). Der Konflikt wird bei Pflegenden verstärkt, die im privaten Umfeld für Menschen sorgen, die ein Risiko haben, schwere COVID-19-induzierte Krankheitsverläufe zu bekommen. „*Im privaten Bereich habe ich pflegebedürftige Schwiegereltern, die ich momentan nicht sehen kann, um sie nicht womöglich anzustecken. Man bewegt sich in Arbeitsquarantäne zwischen Zuhause und Arbeit*“ (KE14). Die Situation eines Abwägens zwischen beruflichen und persönlichen Anforderungen verstärkt moralische Konflikte.

Solche Konfliktsituationen werden durch die eigene Gefährdung aufgrund unzureichender Hygiene- und Schutzausrüstung verstärkt, denn „*Die Gesundheit wird massiv geschädigt. Und weiterhin wird an allen Ecken gespart. Auch an Schutzausrüstung. Desinfektionsmittel wurde durch Chlor und Essig ersetzt, FFP2-Masken müssen mehrere Tage durchgetragen werden, und das Arbeitsschutzgesetz wird gar nicht mehr eingehalten. Pausen ständig gestrichen, Überstunden bleiben unbezahlt, Pausen von der FFP2-Maske gibt es gar nicht, und auch die Arbeitszeitregelungen werden nicht eingehalten*“ (JC155)*. *Das moralische Dilemma besteht darin, dass sich Pflegende beruflich verpflichtet sehen, die Pflegebedürftigen zu versorgen und das Bedürfnis haben, „*seinen Bew. *[Bewohner*innen]* in der aktuellen COVID-Situation beistehen *[zu wollen]* und sie nicht auch noch alleine lassen möchte*“ (KD29), aber auch die Verantwortung zu tragen, sich selbst zu schützen.

Ebenso kann die ständige Anforderung, Hygiene- und Schutzmaßnahmen einzuhalten, die für eine sichere Versorgung erforderlich sind, zu belastenden Situationen führen: „*Die Mehrarbeit mit den Schutzmaßnahmen, die man nicht immer so einhalten kann, wie’s vorgegeben wird, z.* *B. bei einem Notfall*“ (BD56). Außerdem ist „*Die Angst, einen COVID Ausbruch auf der eigenen Station zu erleben, wo jeder Patient zur höchsten Risikogruppe zählt, … allgegenwärtig*“ (IB1). Verantwortung für die Pandemieeindämmung zu tragen und der damit verbundene Schutz bei gleichzeitig professionell guter Pflege, erleben die Studienteilnehmenden als gegensätzlich. Der „*Konflikt zwischen dem Fernhalten einer Infektion und der psychischen Gesundheit *[ist]* enorm … die Fragen rund um Familienbesuche, Mittagstisch, Ehrenamtlichenbesuche, Veranstaltungen, selbstständiges Einlaufen etc. trägt häufig stark zur Tagesstruktur und zur psychischen Gesundheit älterer Menschen bei. Nach meinem Empfinden geht es sehr vielen alten Leuten psychisch sehr schlecht, in der momentanen Situation, und trotzdem möchte ich auch, dass die Kontakte gering gehalten werden, weil ich weiß, dass eine Infektion mit dem Coronavirus für viele der alten Menschen tödlich sein kann. Das ist nur sehr schwer auszuhalten, zumal unsere Leistungskomplexe bzw. das, was die Pflegekundinnen zahlen können, i.d.R. eine umfangreiche psychische und emotionale Unterstützung nicht vorsehen …*“ (FC19). Pflegende beschreiben eine Dehumanisierung, wodurch sie ihre Arbeit als belastend erleben. „*Es ist unmenschlich für Pfleger und Gepflegte. Aber das interessiert doch eh’ niemanden. Es gibt bereits Tote, weil die Pflege nicht durchgeführt werden kann, wie sie sollte. Diese werden aber nicht als Tote des gescheiterten Gesundheitssystems anerkannt, weil sie ja krank waren. Und auch unsere Gesundheit als Pflegekräfte interessiert niemanden. Wir werden immer mehr und mehr verheizt. Bis es irgendwann niemanden mehr gibt, der den Job machen will*“ (JC155).

### *„Trauer, Stress und Wut“*

Das Gefühl, dass Pflegebedürftige nicht gerecht behandelt werden, in Kombination mit schlechter Versorgung wie beispielsweise „*Bewohner zahlen immer mehr als 4600 monatlich bei uns. Teilweise am Morgen nur 5 bis 10* *min Zeit*“ (AD51) führt zu Zweifeln an der eigenen pflegerischen Versorgungsleistung. „*Aufgrund meiner Erlebnisse seit Beginn der Pandemie glaube ich, dass viele unserer Bewohner an der mangelnden Pflege, der mangelnden Zeit, der Isolation und somit dem schwindenden Lebenswillen verstorben sind!*“ (AC62) oder „*Ich zweifle langsam an meiner Arbeit; das ist nicht das Pflegeverständnis, was ich mir vorstelle. Und ich zweifle an mir selbst. Was ich alles tagtäglich tue, was nicht, was ich nicht schaffe … und jede Woche neue Maßnahmen, noch weniger Zeit am Bewohner*“ (BD47). Aus Bedenken und Zweifeln, wegen der als unzureichend erlebten Versorgung der Pflegebedürftigen, resultieren ein schlechtes Gewissen sowie eigene Schuldzuweisungen. „*Ich gehe immer häufiger mit einem schlechten Gewissen nach Hause und mache mir Gedanken, was ich hätte anders machen sollen*“ (EC6). Zudem haben Pflegende in der Pandemiesituation das Gefühl „*für alle verantwortlich zu sein*“ (JC158).

Die anhaltende moralisch belastende Situation während der Pandemie geht mit Zweifel, Wut, Trauer, Schmerz, Müdigkeit, Frustration, Schlafstörungen und einem Gefühl von Allein- und Unverstandensein einher, wie dem nachfolgenden Zitat zu entnehmen ist: „*Ich bin sehr aufgebracht, verzweifelt, wütend, traurig, ausgebrannt.* […] *Ich stehe jeden Dienst mit mir selbst im Konflikt, weil ich nicht den Eindruck habe, dass ich gute Pflege leiste. Das tut weh und macht müde. Leider fehlen mir aber auch Lösungsansätze. Ich nehme viel Frust mit nach Hause, schlafe schlecht *[…] *wir müssen vor Ort immer weiter funktionieren, dort interessiert es niemanden, wie es uns mit der Situation geht. Ich bin sehr gespannt, was aus Ihren Studien hervorgeht! Entschuldigen Sie die Negativität, und dass ich diese auch als Ventil genutzt habe, aber so fühlt sich zurzeit meine Realität an. Vielen Dank für Ihre Arbeit!*“ (BD4). Es wird deutlich, dass die Altenpflegenden ihre Belastungen und Gefühle nicht adressieren können.

Auch Infektionsgeschehen in der eigenen Institution fördern negative Emotionen „*145 von 151 Bewohnern infiziert, 40 Bewohner verstorben, eine Kollegin verstorben. Trauer, Stress und Wut*“ (CE31). Pflegende erleiden „*physische und psychische Qualen*“ (ME11) durch „*mehr Stress …enorme körperliche sowie psychische Belastung, ständige Müdigkeit, Antriebslosigkeit …man kann nicht so pflegen, wie man soll und gerne möchte!! Ich habe nach der Arbeit keine Motivation mehr, etwas zu machen oder zu erledigen, meistens keine Zeit mehr für Freunde/Familie*“ (DB7). Die pandemiebedingten körperlichen und psychischen Belastungen wirken bis in das Privatleben hinein. Die Folgen reduzierter Selbstfürsorge und ständiger Stress und Konfliktsituationen sind bei einigen Teilnehmenden schwer, denn das „*Risiko für Depressionen steigt*“ (LC5). Es gibt Teilnehmende, die angeben: „*seit der Pandemie an einer Depression*“ (RC7) zu leiden. Eine Pflegefachkraft schreibt: „*Ich bin innerlich erschöpft. Ich verzichte berufsbedingt auf viele Menschen aus der Familie und meide sie. Ich habe Angst, sie mit COVID-19 anzustecken. Ich leider unter einer Depression und möchte überhaupt nicht mehr zur Arbeit gehen, aber ich MUSS. Ich bin am Ende meiner Kräfte und weiß nicht, wie es weitergehen wird*“. Andere umschreiben Symptome wie „*innerliche Erschöpfung und die seelische Belastung. Ich bin nicht mehr motiviert genug und arbeite, weil ich es muss*“ (BB26). Neben Motivationsproblemen wird auch eine emotionale Distanzierung bzw. „*Verminderung der Empathie*“ (IE21) beschrieben.

Hohe moralische Belastung und ständige ethische Dilemma können dazu führen, dass Pflegende den Gedanken haben, aus dem Altenpflegeberuf auszusteigen, wie die nachfolgenden Zitate zeigen: „*Persönlich bin ich psychisch am Ende, werde nach dieser Pandemiewelle der Pflege den Rücken kehren, da man kaputtgeht, psychisch wie körperlich. Ich kann es nicht mit meinem Gewissen vereinbaren, die Leute in so kurzer Zeit pflegen zu müssen und keine Zeit FÜR den BW *[Bewohner]* zu haben. Für die BW *[Bewohner*innen]* selbst ist es momentan auch nicht viel einfacher, da gerade demente BW nicht verstehen, was passiert und noch trauriger darüber sind, dass niemand mehr zu Besuch kommt, warum sie ‚eingesperrt‘ werden*“ (BC49) oder „*Mehr Stress, keine ausreichenden Ruhezeiten, extrem hoher Krankenstand, Pflegezustand der Bew. *[Bewohner*innen]* ist nicht mehr akzeptabel *[…] *Ich habe gekündigt und gehe mit Rentenabzug in Vorruhestand*“ (MD25). Andere berichten von Unverständnis und Wut über die aktuelle Situation und fühlen sich nicht unterstützt: „*Im Frühjahr wurde noch geklatscht, und jetzt bekommt man Vorwürfe, dass alles nur wegen uns zu ist*“ (MD21). Sie fordern: „*Jetzt sofort muss dringend etwas geschehen, wir sind alle am Ende und können nicht mehr*“ (AD37).

## Diskussion und Limitationen

Die Aussagen der Pflegenden aus der stationären Altenpflege zeigen ein sehr kohärentes Bild der Pflege in Altenpflegeeinrichtungen während der Pandemie. Gleichwohl ist zu sagen, dass die hier berichtete Subgruppenanalyse ohne soziodemografische Angaben der Teilnehmenden ausgewertet wurde. Dies liegt in der Vorgehensweise der COVID-19-Pflegestudie begründet, die einen niedrigschwelligen Zugang zu den Teilnehmenden ermöglichen soll. Infolgedessen fehlt die Kontextualisierung der Daten und der Datengewinnung. Zudem können aufgrund der einseitigen Kommunikationsstruktur dieses qualitativen Surveys keine Rückfragen an die Teilnehmenden gerichtet werden, die für ein tiefes Verständnis von Phänomenen notwendig sind. Die Analyse erfolgt durch die Narrative, wobei die schriftlichen Textproduktionen der Studienteilnehmenden durch die Leitfragen mitkonstruiert sein könnten.

Die analysierten Kategorien „*Dass wir keine Zeit haben, um ordentlich pflegen zu können*“, „*Vereinsamung*“ – „*Das Personal leidet, die Bewohner so zu sehen*“, „*Konflikte mit Angehörigen und Bewohnern *[…] *Andere Berufsgruppen kollidieren mit uns*“, *„Ständige Angst um Patientensicherheit, aber auch um die eigene Sicherheit“ *und „*Trauer, Stress und Wut*“ offenbaren differenziert Kriterien, die zum Entstehen von Moral distress bei den Altenpflegenden in der Pandemiesituation führen. Die Schilderungen der Studienteilnehmenden enthüllen, dass eben nicht die eigentliche Arbeit, also die Pflege und Unterstützung von älteren pflegebedürftigen und vulnerablen Menschen in ihrem Lebensalltag, eine Belastung für die Pflegenden darstellt. Im Gegenteil, diese pflegerische Arbeit ist ein Motivationsfaktor, den die Pflegenden mit einer hohen Sinnhaftigkeit ihres Berufes bewerten. Sie beurteilen ihr fachliches Können und ihre Entscheidungskompetenzen, die eng am aktuellen situativen Pandemiegeschehen und den von ihnen zu versorgenden Pflegebedürftigen ausgerichtet sind, als Professionalität. Moral distress entsteht nach den Aussagen der Pflegenden, wenn sie entgegen ihren professionellen Werten und ihrem Können arbeiten müssen. Die in dem Kapitel Hintergrund benannte Begriffsbestimmung von Moral distress kann auch auf der Grundlage der Ergebnisse dieser vorliegenden Studie spezifischer gefasst werden. Nicht nur das Wissen, um das berufsethische und professionell-fachlich notwendige Handeln und die Verunmöglichung der Anwendung dieses Wissens und der Kompetenzen durch institutionelle und strukturelle Bedingungen (Epstein und Hamric 2009, Kleinknecht-Dolf et al. 2015; Spirig et al. 2014), sondern insbesondere die bewusste Wahrnehmung dieses Widerspruchs führt zu einem Belastungserleben, wie Corley et al. (2005) ausführen. Morley et al. (2019) spezifizieren dies, indem sie argumentieren, dass das Erleben eines moralischen Ereignisses, das Erleben von „psychischer Belastung“ und eine direkte kausale Beziehung zwischen beiden Faktoren notwendig und hinreichend für das Phänomen Moral distress sind. Das psychische Belastungserleben ist in der Kategorie *„Trauer, Stress und Wut“* eindrücklich von den Pflegenden dargestellt worden. Die körperlichen und psychischen Auswirkungen auf die Pflegenden werden, neben dem benannten Stress und der Wut, teilweise mit einer hohen innerlichen Erschöpfung und Müdigkeit beschrieben. Durch die angewendete Methodik können die Aussagen der Pflegenden über ein eigenes Depressionsgeschehen nicht differenziert werden, das heißt, sie können nicht eindeutig dem Phänomen Moral distress zugordnet werden, da sie auch als Auswirkung von Moral distress gedeutet werden können. Offensichtlich ist gleichwohl, dass das moralische Belastungserleben psychische Auswirkungen hat, die für einige Pflegende nur durch die Aufgabe des Berufs zu vermeiden sind.

Aufgrund der vorliegenden Ergebnisse kann geschlussfolgert werden, dass Moral distress durch

- die Zeitlimitationen aufgrund von Personalmangel, die Pflegende zwingen, pflegerische Tätigkeiten zu rationieren,
- das körperliche Interaktionsverbot und die Interaktionsverhinderung mit Klient*innen, was die therapeutische Beziehungsgestaltung behindert und sogar blockiert,
- die Bewertung von An- und Zugehörigen als Risiko und nicht mehr als Ressource für die Pflegebedürftigen,
- die Funktionalisierung der Pflegenden als Arbeitskraft, die selbst ihre familiäre (Care)Arbeit einschränken muss, um weiterarbeiten zu können, und
- die fehlenden Möglichkeiten, administrative Entscheidungen im Sinne der eigenen professionellen Einschätzungen beeinflussen zu können und die eigene Stimme erheben zu können,

entsteht.

Die vorgestellten Daten zeigen, dass ein notwendiger Faktor für die Entstehung von Moral distress das psychische Belastungserleben ist. Dieses entsteht durch die Wahrnehmung des Widerspruchs zwischen den eigenen professionellen Maßstäben und der durch die strukturellen Faktoren der Pandemie bedingten Unmöglichkeit, diese zu realisieren.

Es kann durch die verwendete Methode keine dezidierte Aussage darüber getroffen werden, ob die Erfahrungen von Moral distress in der Altenpflege Unterschiede zu Zeiten vor der Pandemie aufweisen. Gleichwohl sind bestimmte Faktoren, wie das körperliche Interaktionsverbot, die Veränderungen der Bewertung von An- und Zugehörigen und die berichtete zunehmende Funktionalisierung von Pflegenden, zusätzliche Faktoren, die durch die Pandemiesituation entstanden sind.

Zur Prävention und zur Bewältigung von Moral distress sollte eine langfristige Erneuerung institutioneller Strukturen unterstützt werden. Es gilt, strukturelle und fachliche Lösungen zu entwickeln, die Pflegenden ermöglicht, ihre eigenes Arbeitsumfeld mit partizipativ erarbeiteten Richtlinien, beispielsweise Leitlinien für die Festlegung von Prioritäten (Miljeteig et al. 2021), zu gestalten und pflegerisch fachliche Entscheidungen bei den Pflegenden zu belassen, die die Pflege durchführen. Beispielsweise führt die Übernahme von pflegefremden Aufgaben, wie die Besuchertestung und -dokumentation und die anschließende Desinfektion zu zeitlich notwendigen Rationierungen in der eigentlichen Pflege, die die Versorgungsqualität erniedrigen und die Pflegende belasten. Hier sollten Assistenz- und/oder Ehrenamtsmodelle entwickelt werden. Auch die Entscheidungen zur Reduktion oder Aufrechterhaltung von Besucher*innenkontakten sollten situativ mit den Pflegenden getroffen werden, die die betreffenden Bewohner*innen versorgen und die die Lebenswelt und damit die Hoffnungen, Ängste und Pflegebedarfe der Pflegebedürftigen einschätzen können. Versorgungs- und Betreuungsrichtlinien sollten unter Bezugnahme auf die, von den Pflegenden erstellte, Pflegeplanung mit den Pflegenden partizipativ entwickelt werden.

Des Weiteren wird eine transparente und kontinuierliche Kommunikation zwischen Pflegenden und Führungskräften als eine zentrale Strategie verstanden, um das Auftreten von Moral distress zu minimieren oder überhaupt erst bewältigen zu können (Villa et al. 2021). Ethische Fallbesprechungen und Supervision können dazu beitragen, dass sowohl die Versorgungsqualität als auch die Belastungssituation für die Pflegenden so verbessert werden, dass das Erleben von Moral distress verringert werden kann.

Dazu benötigen Pflegende gleichwohl autonome Entscheidungskompetenzen und hohe fachliche Kompetenzen, was eine qualitativ hochwertige Ausbildung und eine beträchtliche Fachkraftquote bedeutet.
